# Personally relevant online advertisements: Effects of demographic targeting on visual attention and brand evaluation

**DOI:** 10.1371/journal.pone.0212419

**Published:** 2019-02-15

**Authors:** Kai Kaspar, Sarah Lucia Weber, Anne-Kathrin Wilbers

**Affiliations:** Department of Psychology, University of Cologne, Cologne, Germany; State University of New York Downstate Medical Center, UNITED STATES

## Abstract

Global investments in online advertising rise quickly but internet users often avoid looking at ads due to established banner blindness. Demographic targeting is expected to overcome this tendency by attracting users’ attention to more self-relevant ad content. However, little is known about the effect of demographically targeted versus non-targeted ads on users’ actual attention allocation during exposure to webpages. The present study aimed to further fill this empirical gap by clarifying whether demographic targeting attracts visual attention and to exploratively examine whether it also affects brand attitude and website evaluation, as suggested by previous studies. Eye tracking data revealed that demographic targeting can have medium- to large-sized effects on several eye movement parameters when internet users are in a free-viewing mode. In contrast, demographic targeting did not influence brand attitude and website evaluation. We conclude that attention for personally relevant advertisement can be strong. However, attention, although being a necessary condition for subsequent judgment formation according to the model of human information processing, is not sufficient to elicit positive effects at the level of subjective judgments.

## Introduction

Online advertising is a hot topic nowadays and the overall global investments in online advertising rose quickly to about 230 billion dollars in 2017 [1]. The key question is whether the effects are worth the costs. According to general advertising models, the effectiveness of online advertisements can be evaluated regarding the processing stage (e.g., attentional or mnemonic effects), communication effects (e.g., users’ attitudes towards brands), and user behavior (e.g., click-rates) [2]. However, internet users have learned to partially avoid looking at online advertisements on webpages, establishing a phenomenon called “banner blindness” [3]. Thereby “ads are more likely to be cognitively avoided since it is an automatic, subconscious process that occurs in parallel with the browsing activity and does not require any behavioral action by the consumer” ([4], p. 51). The extent to which internet users actually avoid ads is part of an ongoing debate, as this conclusion has occasionally been derived from indirect measures such as memory tests [4] or users’ self-reports [5]. In contrast, Hervet at el. [6] used eye-tracking methodology and found that users actually do fixate banners, but the number of fixations and the fixation duration on banners are way smaller than on areas with editorial content. Similarly, Hamborg et al. [7] found that internet users generally fixated banner ads during an information search task, but static ads were less often fixated than animated ads. This strong tendency to reduce one’s own exposure to ads or even to avoid looking at them completely may be explained by the substantial amount of online advertisements being unrelated to the needs of the individual internet user, leading to “increasing advertising saturation levels” ([8], p. 1). Hence, marketers as well as scientists search for design factors which help to overcome this problem [9].

Indeed, creating attention for advertisements is a challenging but also the most fundamental task in marketing. As outlined in the model of human information processing by Tam and Ho [10], attention to a web stimulus is always the first stage of the processing stream. Attention mediates the effects of ads on memory, attitudes, judgments, and behavioral responses. Advertisements that are not noticed remain ineffective. Besides some rather intrusive variants of ads, like pop-up ads which were found to create enhanced feelings of psychological reactance in internet users [11], increasing the personal relevance of online ads appears to be a promising strategy to attract users’ visual attention. From a theoretical perspective, ads that are personally relevant should be attended by internet users and hence facilitate cognitive processing (cf. [12]). Personal relevance leads to the experience of involvement which motivates attentional and comprehension processes [13], and personal relevance is associated with higher approach motivation as indicated by neurophysiological findings [14]. According to the selection-for-action account [15], sensory information (e.g., an advertisement) is considered with respect to its relevance for future actions. Given that gaze behavior significantly depends on the agent’s motivational state (cf. [16, 17]), more versus less personally relevant advertisements are more closely related to needs of customers and hence should motivate them to visually explore the advertisements. In this sense, personal relevance of ads may be the key to overcome the tendency for banner blindness.

Researchers have investigated the effectiveness of numerous forms of ads leading to an increased personal relevance for the users. This also includes personalized advertising via e-mails, letters, and telephone calls [18]. With respect to webpages, there are several methods used to personalize or customize advertisements. A widespread account is the analysis and specification of general characteristics shared by the target group of an advertising campaign. This “demographic targeting” focuses on the demographic properties of the users–such as gender, age, or place of residence–in order to tailor the ad content to the target group [19]. Based on the analysis of users’ demographic group membership, marketers and retailers get insights into the needs and interests likely shared by the members of the target group. The concept of demographic targeting is based on the theory of social categories postulating that similar people act in a similar way [20]. In this sense, demographic targeting provides a somewhat generic, but basically interesting framing of an advertisement with respect to the target group. Importantly, in contrast to strategies based on the collection and analysis of extensive user date in order to personalize ad content to the idiosyncrasies of the individual user, demographic targeting barely activate privacy concerns. In general, the extent to which personal data is used in advertising may reduce users’ purchase intention [21] and increase privacy concerns [22, 23].

Demographic targeting has attracted the interest of online marketers in a variety of areas [24], and many practitioners are interested in and willing to pay more for demographically targeted advertising [25]. It has been shown that the estimation of advertising effects in specific product areas becomes more accurate when demographic factors are considered [26]. However, empirical research that examines true attentional effects of demographically targeted ads on webpages is surprisingly sparse. In particular, previous studies commonly used self-reports or behavioral measurements, such as click-rates, as indicators of attention allocation after the exposure to advertisements. Little is known about users’ actual attention allocation *during* their exposure to webpages with respect to the effect of more versus less personally relevant ad content. According to the “eye-mind hypothesis” [27] eye movements provide a dynamic trace of one’s attention and cognitive focus. To our best knowledge, only one study published in English language has applied eye-tracking methodology so far in order to compare the effectiveness of demographically targeted versus non-targeted ads on internet users’ visual attention: Köster et al. [19] presented 16 webpages with either demographically targeted or non-targeted ads of different brands while tracking participants’ gaze behavior during an information search task. Demographic targeting was based on participants’ gender, age, professional interest, place of residence, and occupation. Participants showed significantly more entry fixations on the slogan and logo (but not picture) area of the demographically targeted versus non-targeted version of the ads, but the effect sizes were rather small (below *d* = .20). Nonetheless, post-experimental recognition performance for diverse ad elements was substantially increased when embedded in demographically targeted ads. Small effects on the level of attention can apparently elicit substantial effects on the level of memory performance.

However, it is important to have a closer look to the kind of the primary task participants had to perform during webpage exposure: Köster and colleagues [19] used an information search task, but some previous research suggested that online advertisements are less effective in the case of goal-directed searching compared to a free-viewing mode [28, 29]. Hamborg et al. [7] concluded “that banner ads have little or no impact if users are in an information-seeking mode” (p. 577). Although Bang and Wojdynski [30] similarly argued that “people who freely navigate web pages have greater cognitive capacity available for non-target information (e.g., advertisements) than those who seek specific information” (p. 869), they found a strong effect of personalized ads (by means of participants’ name and individual geographic information) versus non-personalized ads on visual attention only in subjects who were engaged in an information search task, whereas participants in a free-viewing mode showed only weak attention to the ads and no effect of personalization. The authors speculated that “when people are not strongly engaged and involved in the media consumption process on the web, they tend to show habitual and automatic ad avoidance phenomenon for both personalized and non-personalized ads, a phenomenon called ‘banner blindness’” (p. 873). As a consequence, and with respect to demographic targeting, it remains open whether we would observe stronger effects of demographically targeted ads on users’ visual attention in a free-viewing scenario compared to the small effects found by Köster et al. [19] who used an information-search scenario, as suggested by several studies on the effect of the user task on visual attention [7, 28, 29]. Alternatively, the attentional effect of demographic targeting may even completely disappear, as suggested by the finding of Bang and Wojdynski [30]. We hence tested the following hypothesis in a two-tailed manner:

Hypothesis 1: *Demographically targeted versus non-targeted advertisements in the form of display ads differ in their impact on webpage users’ visual attention*.

With respect to general advertising models [2], Köster et al. [19] exclusively focused on the processing stage of advertisements, that is, effects on visual attention as well as memory effects assessed by the recognition performance for ad content after webpage exposure. In order to expand our knowledge about the effects of demographic targeting in advertising, we further examined the attentional process but shifted the focus from memory to communication effects in terms of users’ attitudes towards the advertised brands. We were not interested in users’ evaluation of the ad itself, because the specific written and pictorial content of an ad often varies across different ad versions of a brand. Products, slogans, and pictures depicted are often only prototypical representatives of a product class or a whole range of ad designs. For example, a publisher usually offers a wide variety of literature, a cosmetics brands is characterized by numerous product types, and some brand logos are accompanied by varying images and slogans tailored to the advertising context. However, while substantial literature on brand evaluation exists, the potential effect of demographic targeting on brand evaluation has not been examined so far. Hence, this part of the study was explorative in order to shed first light on this issue.

Nonetheless, some rationale and hints for an effect of demographic targeting on brand evaluation exist. On the one hand, a few studies have already shown effects of the personal relevance of ad content on ad evaluation, but without considering demographic targeting: The personal relevance of an advertisement has been found to be positively correlated with users’ attitude towards the ad [31]. Moreover, van Doorn and Hoekstra [21] found that purchase intentions for an advertised product increased when the ad content fitted personal needs, but only if the intrusiveness of the ad was low in terms of personalization via using the user’s name and transaction information. As demographic targeting does not incorporate information of the individual user, a positive effect of demographic targeting on brand evaluation is likely. Finally, Kim and Han [32] found that the personal relevance of advertisements was also positively associated with perceived credibility and entertaining potential (hedonic value). On the other hand, research has provided support for a strong direct and indirect (via brand cognitions) influence of attitudes towards an ad on brand attitudes [33, 34]. Related to this, Muehling [35] found that in the context of comparative advertising, where multiple brands are referenced and displayed, the transfer of affect from ads to brands occurs almost exclusively for the sponsoring brand. Given the positive effects of ad relevance on attitudes towards the ad on the one hand, and the strong positive relationship between ad attitudes and brand attitudes on the other, we exploratively examined the following research question:

Research Question 1a: *Do demographically targeted versus non-targeted advertisements lead to a more positive evaluation of the advertised brand in terms of the users’ overall attitude towards the advertised brand*, *interest in the brand’s products*, *purchase intention*, *the brand’s hedonic value*, *and brand trust*?

Additionally, Gierl and Bambauer [36] found an interesting halo effect: The evaluation of specific elements of websites of seven international car manufacturers (e.g., reports about motorsport events and test reports) was also positively related with the evaluation of the whole website. Halo effects are defined as a systematic cognitive but unconscious bias of making generalized judgements based on one salient piece of information [37]. Indeed, it is conceivable that a positive evaluation of the advertised brands elicited by demographic targeting my also affect the evaluation of the website. We thus exploratively examined the following research question:

Research Question 1b: *Is a* w*ebsite that contains demographically targeted versus non-targeted advertisements evaluated more positively regarding its overall appeal*, *hedonic qualities*, *and pragmatic quality*?

## Method

### Ethics statement

This study was approved by the Ethics Committee of the Medical Faculty of the University of Cologne (No. 15–151) and all participants gave written consent.

### Participants

Following Köster et al. ([19]: *n* = 48), our sample comprised 49 female psychology students from the University of Cologne (*M*_*age*_ = 21.24, *SD*_*age*_ = 2.89). To mesh with demographic targets we used in the advertisements, participants were selected according to the same five demographic properties used by Köster et al.: Gender (female), age (18 to 28 years), professional interest (psychology), place of residence (Cologne, Germany), and current occupation (student). This set of variables covered some of the most important demographic properties usually reported and examined in order to specify sample characteristics (cf. [38, 39, 40]). Participants were randomly assigned to the two advertisement conditions, namely demographically targeted ad content (*n* = 26) or non-targeted ad content (*n* = 23).

### Procedure

The experiment was conducted under standardized conditions in a laboratory at the University’s campus. Upon arrival, the participants were introduced into the study procedure, but they were not aware of the demographically targeted advertisements. Next, all participants performed and passed the Ishihara Test for Color Blindness [41]. Participants were told to freely observe webpages of a newly designed online magazine for students. This scenario was selected according to Kaspar et al. [42] as it provides a plausible cover story and effectively elicits a free-viewing mode [43, 44].

Participants observed 25 webpages presented in randomized sequence. In line with previous studies (e.g., [19, 42, 44, 45, 46]), the participants did not interact with the webpages and exposure time was fixed to avoid possible confounding effects. Each webpage included a different advertisement which was either targeted to the participants’ demographic properties, or not. The viewing time for each webpage was 45 seconds in order to match the presentation duration realized by Köster et al. [19], enabling a direct comparison of the total ad observation time. This viewing time was pre-tested by Köster et al. and the authors of the present manuscript to optimally fit the complexity of the used webpages.

After the eye-tracking session, participants evaluated the website by means of the AttrakDiff 2.0 questionnaire [47] measuring the website’s overall appeal as well as hedonic and pragmatic qualities. Finally, participants evaluated the brands shown in the advertisements on four dimensions: Interest in the brand’s products, purchase intention, the brand’s hedonic value, and brand trust.

### Apparatuses

All stimuli were presented on a 22-inch display with a resolution of 1680 x 1050 pixels and a refresh rate of 60Hz. The distance between display and head was 70cm. Eye movements were recorded by a remote SMI RED 500 Eye Tracker (SensoMotoric Instruments) that uses infrared pupil tracking at a sampling rate of 500Hz and compensates for head movements. To calibrate, participants made saccades to a grid of nine fixation spots on the screen, which appeared one by one in a pseudo-randomized order. Tracking of the eye which provided the lower validation error started as soon as this value was below 1° visual angle. Each webpage was preceded by a fixation cross for a variable duration of 1.0 to 1.5 seconds. Saccade detection was based on a peak velocity threshold of 40°/s and a minimum saccade duration of 22ms. The minimum duration for valid fixation was 50ms. The first fixation of each trial was excluded from the analyses since its localization was an artifact of the preceding fixation cross. Recorded data were pre-processed using the SMI BeGaze Software and exported to SPSS 23.0 (IBM, Inc.) for statistical analyses.

### Materials

#### Stimuli

**Fig 1:**
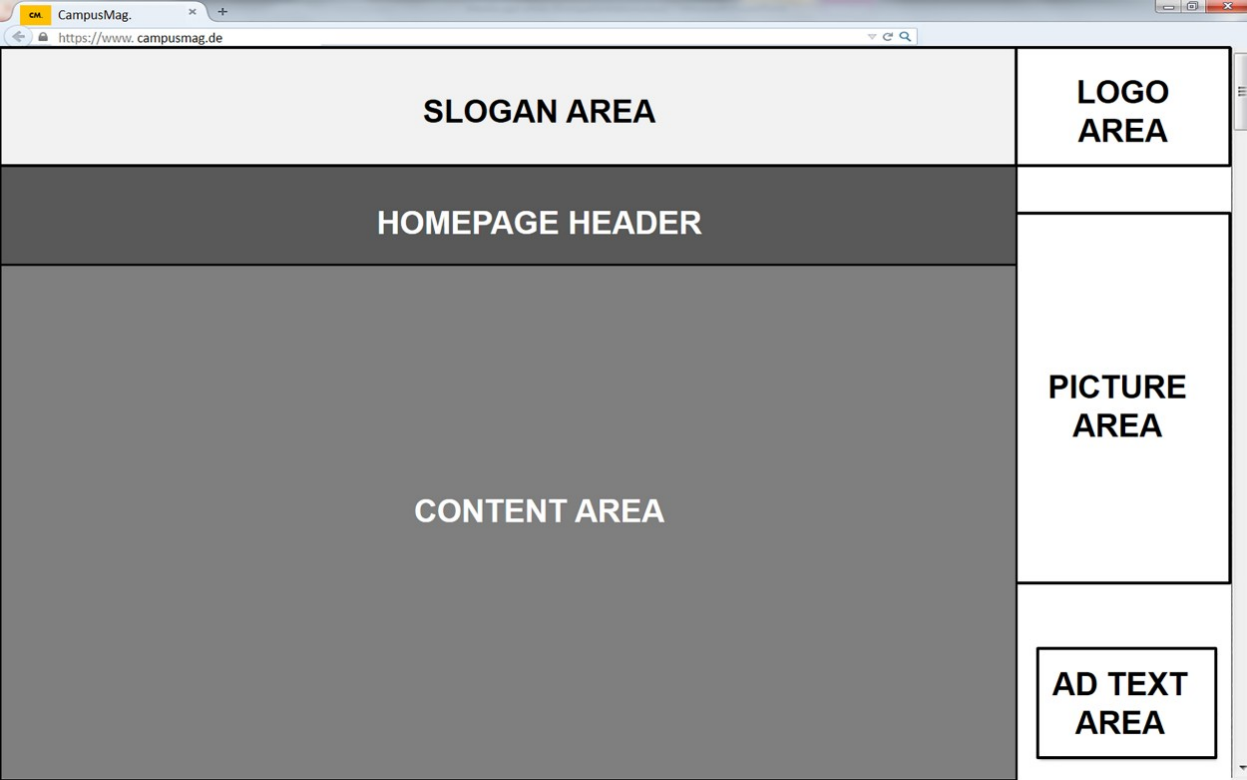
The schematic structure of all webpages used in the present study. The advertisement comprised a super leaderboard banner (slogan area) and a wide skyscraper banner (white area including the logo of the brand, a picture, and an offer-related text).

In favor of direct comparability we reproduced the visual design created by Köster et al. [19], that is, the advertisements were constituted by a super leaderboard banner and a wide skyscraper banner (cf. [48]). This design reflects a standard format of advertisements in Germany as common, for example, in the online version of Germany’s most sold newspaper “Bild Zeitung” (over 1,6 Mio. sold copies in 3^rd^ quarter 2018, [49]). As shown in Fig 1, the leaderboard banner was horizontally placed on top of each webpage and contained a brand-related slogan of a few words (*slogan area*). The skyscraper banner was placed on the right-hand side of the webpages; it contained the logo of the brand (*logo area*) and a picture of one of the brand’s products or an image related to the slogan (*picture area*), subtitled by a short offer-related ad text (*ad text area*). The leaderboard banner and the skyscraper banner constituted the *full advertisement area*. The editorial content area included several images, each accompanied by a short abstract of the full text (accessible via a displayed but non-active link). Webpages were static and participants did not scroll.

Overall, we created 25 webpages in two versions each (demographically targeted versus non-targeted ad). The whole procedure followed Köster et al. [19]: Demographic targeting was manipulated by using the same brand but slightly changing the slogan (slogan area) and the picture (picture area) of the advertisement, while the logo area and the ad text area remained unchanged. Demographic targeting addressed the five demographic properties of the sample: Gender (female), age (18 to 28 years), professional interest (psychology), place of residence (Cologne, Germany), and current occupation (student). In order to enhance the personal relevance for the group with demographic targeting, the advertisements referred to women, psychology students, young people, and Cologne residents, whereas in the control group without demographic targeting the advertisements referred to, for example, men, elderly people, computer science students, or Berlin residents.

We used 25 real brands which were expected to be both desirable and familiar to all participants. We conducted a pre-test with five persons from the target group specified by the demographic properties above (not included in the final sample). They were independently asked whether the brands are familiar to them. All five persons correctly recognized (yes vs. no) all brands on the basis of the corresponding banner ad. As shown in Table 1, this set covered a large range of different brands and related product classes in order to counterbalance potential inter-individual differences in product involvement which is defined as the perceived relevance of a product class based on the inherent needs, interests, and values of the individual consumer [50].

**Table 1:** The set of brands, corresponding product classes, and associated demographic properties.

Brand	Product class	Demographic property
Balea	Lip balm	Gender
AOK	Health insurance	Occupation
Springer	Books	Professional interest
Colgate	Toothpaste	Age
Fitness First	Gym membership	Place of residence
Nivea	Hair styling gel	Gender
Deutsche Bahn	Travelcard	Occupation
Pearson Education	Literature	Professional interest
L‘Oréal	Facial creme	Age
Luups	Voucher book	Place of residence
Schauma Schwarzkopf	Shampoo	Gender
T-Mobile	Mobile phone contract	Occupation
Stepstone	Tickets for career day	Professional interest
Theater im Bauturm	Theater tickets	Age
Marco Polo	Guide for recreational activities	Place of residence
Wilkinson Sword	Shaving kit	Gender
Microsoft Office	Software	Occupation
GEO	Magazine	Professional interest
Parship	Online dating portal	Age
Airberlin	Airline tickets	Place of residence
Rexona	Antiperspirant	Gender
Axa	Liability insurance	Occupation
Dove	Shampoo	Age
Zeit Campus	Magazine	Professional interest
Drive Now	Car sharing	Place of residence

In order to realize demographic targeting in the ads, the slogan was manipulated by altering few words, for example “The shampoo for *women/men*” (gender) or “Perfectly prepared for the study of *psychology/geology*” (professional interest). We correspondingly manipulated the picture area by changing the product’s characteristics or the image related to the slogan, for example a psychology (demographically targeted) versus geology (non-targeted) text book, a shampoo bottle with a female (demographically targeted) versus male (non-targeted) face on its cover, or an image of a young (demographically targeted) versus older (non-targeted) woman.

Importantly, the visual properties of the demographically targeted advertisements and their non-targeted counterparts only differed slightly. They were matched regarding their visual saliency to exclude biases in eye movements which are elicited by systematic differences in basal image properties (cf. [19, 42, 51]). We separately controlled the image properties of the leaderboard banner and the skyscraper banner. For this purpose, each banner was analyzed by means of two saliency computations before the study, namely the standard algorithm by Itti et al. [52] predicting fixation locations by a decomposition of images into several physical features (e.g., contrast and orientation) and a more recent graph-based visual saliency algorithm by Harel et al. [53]. We applied both algorithms to our final stimulus set and found no difference in visual saliency between demographically targeted and non-targeted ads regarding the leaderboard banner, both *Z*s ≥ -0.31, *p*s ≥ .757, and the skyscraper banner, both *t*s(24) ≤ 0.69, *p*s ≥ .500. Importantly, the two procedures applied do not compare real fixations on stimuli. Instead, the two computational models *a priori* analyze visual stimuli regarding numerous pictorial components and nicely predict the probability of specific fixation locations on the basis of the given image statistics. More specifically, the algorithm by Itti et al. [52], for example, analyzes multiscale image features and combines them into a single topographical saliency map. The purpose of this saliency map is to represent the conspicuity (saliency) at every location of the visual stimulus by a scalar quantity and to predict the selection of attended locations, based on the spatial distribution of saliency. The models applied here are heavily used in the field of vision research and are suitable to control for artificial results in fixation behavior that may result from systematic biases in image statistics between experimental groups. That is, if we would find differences regarding real fixations between demographically targeted and non-targeted ads, this effect would be solely driven by the ads’ semantic content (reflecting the degree of personal relevance) and not by differences on the level of basal image statistics. Most of the previous studies on the effect of personalized advertising on visual attention did not control for biases in image statistics (e.g., [30, 54]) and hence may have over- or underestimated the impact of the semantic content on attention.

#### Brand evaluation

Participants’ were presented all brand logos embedded in the advertisements (logo area) in randomized sequence. They were asked to rate each brand on four dimensions (English translations of German items): To measure brand trust, we used the item “*I trust this brand*” borrowed from Chaudhuri and Holbrook [55]. Following Hur et al. [56], we used the item “*I like this brand*”to assess the brand’s hedonic value. Purchase intention was measured by the item “*It is likely that I will buy a product of this brand in the future*” (cf. [57]). Participants’ interest in the brand’s products was assessed by “*I am interested in the brand’s products*” [58]. We used a 7-point rating scale for each item (1 = *strongly disagree*; 7 = *strongly agree*).

#### Website evaluation

Participants evaluated the whole website by means of the AttrakDiff 2.0 questionnaire [47]. This instrument assesses the overall appeal, the hedonic qualities “identification” and “stimulation”, as well as the perceived pragmatic quality of interactive products such as websites. Each of these four concepts is operationalized by seven word pairs on a 7-point semantic-differential scale. The appeal scale assesses the global attractiveness of the website (e.g., motivating versus discouraging). The hedonic quality is split into two aspects: “Identification” describes the possibility to communicate a desirable identity to others (e.g., isolating versus coupling), while “stimulation” assesses the amount to which the website supports striving for personal development (e.g., original versus conventional). The pragmatic quality is an assessment of the website’s perceived usability (e.g., simple versus complicated).

### Data analysis

#### Eye movements

We extracted five parameters from participants’ eye movements: *dwell time* (i.e., the time participants fixated a specific area of interest, AOI), *number of fixations* (the total number of fixations within an AOI across the whole trial), *entry time* (the time stamp of the first saccade to AOI), *number of entry fixations* (first entry into the AOI plus all further saccadic jumps from outside elements to AOI), and *mean fixation duration* (the mean duration of individual fixations). The AOIs were defined by the advertisement areas described above (see Fig 1). We considered the varying size of the picture area across advertisements when defining corresponding AOIs.

#### Brand evaluation

We analyzed the effect of demographic targeting on brand trust, the brand’s hedonic value, purchase intention, and participants’ interest in the brand’s products. We also computed a composite score serving as an indicator of participants’ overall attitude towards the brand (coefficient alpha reliability α = .89).

#### Website evaluation

Before the statistical analysis, we averaged across the seven items measuring the website’s overall appeal (α = .87), its hedonic qualities “identification” (α = .69) and “stimulation” (α = .88), as well as its perceived pragmatic quality (α = .69). The original 7-point scale (ranging from 1 to 7) was rescaled during the statistical analysis (-3 to +3) to facilitate the interpretation of absolute values (cf. [59, 60]).

## Results

### The effect of demographically targeted advertisements on visual attention (Hypothesis 1)

We initially compared the demographically targeted and the non-targeted advertisements regarding the five eye movement parameters. If eye movement parameters were not normally distributed (Kolmogorov-Smirnov test with Lilliefors correction: *p* > .10), we calculated Mann-Whitney *U*-tests (*Z*-statistic) instead of *t*-tests for independent samples. Statistical analyses were conducted with IBM SPSS Statistics (Version 23). As shown in Table 2, we found strong support for Hypothesis 1: Demographically targeted ads attracted more visual attention in terms of dwell time and number of fixations. Thereby, this effect reached statistical significance on all areas of the advertisement, except the logo area. Moreover, the mean duration of fixations was substantially longer on the picture area of demographically targeted ads, whereas no effect was found regarding the slogan area, the ad text area, and the logo area. Effect sizes of statistically significant effects were medium to large according to common rules of thumb [61]. In contrast, the point of time when participants made their first saccade to the elements of the advertisement (entry time) was not significantly affected by demographic targeting and occurred relatively late (16–28 seconds) after webpage onset. Finally, the slogan area of demographically targeted ads was more frequently (re-)visited as indicated by the number of entry fixations. Consequently, the results clearly support the effectiveness of demographic targeting at the level of visual attention.

**Table 2:** Effects of demographically targeted versus non-targeted advertisements on visual attention.

	Demographically targeted advertisements	Demographically non-targeted advertisements			
	*M* (*SD*)	*M* (*SD*)	*t / Z*	*p*	*d*
**Dwell time (ms)**					
Full advertisement area	3819.99 (1398.97)	2500.99 (1405.46)	*t* = 3.287	.002**	0.941
Slogan area	1168.55 (547.20)	786.18 (532.19)	*t* = 2.473	.017*	0.708
Wide skyscraper area	2246.58 (842.05)	1428.98 (814.01)	Z = -3.185	.001***	0.986
- Logo area	275.56 (238.82)	181.00 (160.29)	Z = -1.723	.085^+^	0.459
- Picture area	1057.79 (482.70)	700.32 (424.28)	*t* = 2.737	.009**	0.783
- Ad text area	802.88 (428.13)	530.77 (338.49)	*t* = 2.445	.018*	0.700
**Number of fixations**					
Full advertisement area	15.91 (6.38)	11.11 (6.59)	Z = -2.444	.015*	0.741
Slogan area	5.85 (2.78)	4.07 (2.75)	*t* = 2.243	.030*	0.644
Wide skyscraper area	8.82 (3.54)	6.13 (3.66)	*t* = 2.613	.012*	0.748
- Logo area	1.32 (0.89)	0.99 (0.82)	Z = -1.553	.120	0.385
- Picture area	3.99 (1.63)	3.02 (1.69)	*t* = 2.056	.045*	0.585
- Ad text area	3.51 (1.94)	2.47 (1.72)	*t* = 1.962	.056^+^	0.565
**Ø Fixation duration (ms)**					
Full advertisement area	184.11 (36.54)	157.56 (27.68)	*t* = 2.837	.007**	0.812
Slogan area	152.53 (25.37)	142.15 (28.67)	*t* = 1.344	.185	0.385
Wide skyscraper area	204.80 (39.03)	168.32 (31.57)	*t* = 3.566	.001***	1.021
- Logo area	159.13 (45.91)	155.76 (40.05)	*t* = 0.268	.790	0.078
- Picture area	221.79 (49.47)	175.15 (37.68)	Z = -3.666	< .001***	1.052
- Ad text area	193.69 (44.85)	176.74 (43.72)	*t* = 1.324	.192	0.382
**Entry time (ms)**					
Full advertisement area	15914.88 (10179.22)	19532.79 (10169.42)	*t* = -1.242	.220	0.356
Slogan area	17065.93 (10876.83)	20361.16 (10402.38)	*t* = -1.080	.286	0.309
Wide skyscraper area	20595.54 (10283.30)	23064.85 (9301.49)	*t* = -0.877	.385	0.251
- Logo area	23589.08 (10375.45)	23867.62 (9377.42)	Z = -0.062	.951	0.028
- Picture area	21332.06 (10333.38)	22893.02 (9200.99)	*t* = -0.555	.581	0.159
- Ad text area	23495.50 (9235.27)	27756.91 (8204.57)	*t* = -1.684	.099^+^	0.486
**Number of entry fixations**					
Full advertisement area	2.27 (1.24)	2.05 (1.37)	Z = -0.932	.352	0.169
Slogan area	1.39 (0.72)	1.11 (0.89)	Z = -2.175	.030*	0.348
Wide skyscraper area	0.97 (0.50)	0.95 (0.53)	*t* = 0.133	.895	0.039
- Logo area	0.44 (0.34)	0.42 (0.31)	Z = - 0.020	.984	0.061
- Picture area	1.00 (0.42)	0.83 (0.55)	*t* = 1.227	.226	0.350
- Ad text area	0.53 (0.27)	0.56 (0.37)	Z = -0.201	.841	0.094

^+^*p* < .10

*p < .05

**p < .01

***p < .001

### The effect of demographically targeted ads on brand evaluation (Research Question 1a) and website evaluation (Research Question 1b)

We did not find an effect of demographically targeted advertisements on brand evaluation. As shown in Table 3, none of the evaluation dimensions was affected by the manipulation. Also, demographic targeting did not influence participants’ evaluation of the website. Overall, the website was positively evaluated as the mean scale values significantly differed from zero in both the demographically targeted and non-targeted condition (all *p*s < .001), except the evaluation of the hedonic quality “stimulation” (both *p*s > .05). Consequently, although demographically targeted ads attracted more visual attention, this effect was neither paralleled by a more positive attitude toward the brands nor by a better evaluation of the online magazine. Importantly, brand evaluation was based on the presentation of brand logos which, however, did not attract significantly more visual attention when embedded in a demographically targeted advertisement (see above).

**Table 3:** Effects of demographically targeted versus non-targeted advertisements on brand attitude and website evaluation.

	Demographically targeted advertisements	Demographically non-targeted advertisements			
	*M* (*SD*)	*M* (*SD*)	*t / Z*	*p*	*d*
**Brand evaluation**					
Overall attitude towards brand	3.98 (0.63)	3.93 (0.45)	*t* = 0.290	.773	0.090
Brand trust	4.17 (0.67)	4.16 (0.53)	*t* = 0.054	.957	0.016
Brand’s hedonic value	4.22 (0.68)	4.17 (0.54)	*t* = 0.279	.782	0.081
Purchase intention	3.68 (0.77)	3.65 (0.61)	Z = -0.702	.483	0.043
Interest in brand’s products	3.85 (0.73)	3.75 (0.51)	*t* = 0.530	.598	0.157
**Website evaluation**					
Overall appeal	1.54 (0.80)	1.61 (0.73)	*Z* = -0.221	.825	0.091
Hedonic quality “identification”	1.05 (0.65)	1.15 (0.55)	Z = -0.463	.644	0.165
Hedonic quality “stimulation”	0.43 (1.07)	0.33 (0.91)	*t* = 0.347	.730	0.100
Pragmatic quality	1.60 (0.69)	1.73 (0.46)	Z = -0.342	.732	0.219

Note: The scales for brand evaluation ranged from (1 = *strongly disagree*; 7 = *strongly agree*); the scales for website evaluation ranged from -3 to +3.

## Discussion

Personally relevant online advertising is intended to attract the attention of internet users and to counteract the tendency for banner blindness. Demographically targeted ads are self-relevant stimuli which generally facilitate cognitive processing [12], motivate attentional and comprehension processes [13], provoke approach motivation [14], and should guide selective attention due to their relevance for future actions (cf. [15]). However, we only have very limited knowledge about users’ actual attention allocation during exposure to webpages that include demographically targeted versus non-targeted advertisements. The present study aimed to further fill this empirical gap by examining whether demographically targeted advertising attracts more visual attention when internet users are in a free-viewing mode (Hypothesis 1), and whether demographic targeting also leads to a more positive evaluation of advertised brands (Research Question 1a) and of the website in which demographically targeted ads are embedded (Research Question 1b).

### Hypothesis 1: The effect of demographic targeting on visual attention

With respect to the focal hypothesis of the present study, the results draw a clear picture: Demographically targeted ads attracted more attention in terms of fixation number and dwell time on diverse banner areas, except the area in which the brand logo was depicted. Here, the effect did not reach statistical significance, but the descriptive statistics show that the logo attracted at least a bit more attention when embedded in a demographically targeted ad. This latter finding might be discouraging for marketers as it may impede the consolidation of brand loyalty. Also, pictures embedded in the ads elicited longer fixations when being targeted to the demographic properties of the user group, supporting Hypothesis 1. These effects were characterized by a considerable effect size ranging from *d* = .57 to 1.05. In contrast, Köster et al. [19] only found effect sizes below *d* = .20 when a demographically comparable sample of participants performed an information search task. This result supports previous observations according to which banner ads only have a small impact when participants are searching for specific information [7]. Apparently, browsing through webpages releases greater cognitive capacity available for non-target information such as advertisements (cf. [30]), leaving more room for effects of personally relevant advertisements on users’ attention. This conclusion is in line with previous research indicating that online advertisements are less effective in the case of goal-directed searching compared to a free-viewing mode [28, 29].

However, the fact that our data did not show significant effects of demographic targeting on initial fixation time (entry time) indicates that ad content did not hit vision from the outset. In fact, it takes some time to recognize that an ad is personally relevant and its effect on visual attention usually occurs with a temporal delay [7, 19]. Importantly, participants spent, on average, 3.20 seconds (*SD* = 1.54) on the full banner area while webpages were presented for 45 seconds each. This quantity does not indicate banner blindness (cf. [3]). Instead, we might consider it as a substantial portion of attention allocation because there was much more to explore on each webpage presenting several written abstracts of current “hot topics” for female students (e.g., best travel destinations for students, new opportunities for student living, new dating apps, financial support for students etc.). However, it should be noted that the inter-subject variance in the eye movement parameters was rather big. Although participants spent substantial time on the ads on average, they visually exploited the ad content with varying depth.

To conclude, although demographic targeting provides a somewhat generic framing of online advertisements that is not tailored to the specific need configuration of individual users, it nonetheless is an effective method to increase, on average, the target group’s visual attention to the advertisements. Apparently, the high interest of online marketers in demographic targeting [24] is quite reasonable as well as their willingness to pay more for demographically targeted advertising (cf. [25]). However, and with respect to general advertising models [2], this effect only addresses the processing stage, whereas the effectiveness and value of advertising campaigns are also measured by effects at the communication and the behavioral level. For this reason, we additionally examined participants’ brand and website evaluation.

### Research Question 1: No effect of demographic targeting on brand and website evaluation

In contrast to the attentional effect, demographically targeted ads did not lead to a more positive evaluation of the advertised brands in the present study. At least some previous studies [10, 32] found that personally relevant online advertisements were associated with positive attributes. We see at least three alternatives for the absence of an effect in the present study: (1) Either demographic targeting did not affect participants’ attitudes towards the ads (not tested), and thus the usual transfer to brand attitudes could not occur [33, 34; 35], (2) or positive ad evaluation does not necessarily generalize to the advertised brand although extensive research suggests both a strong direct and indirect effect of ad attitudes on brand attitudes (cf. [33, 34]), (3) or the absence of an effect of demographic targeting was actually linked to visual attention, because brand logos–serving as stimuli in the brand evaluation task–did not attract significantly more visual attention when embedded in demographically targeted (versus non-targeted) advertisements. We cannot make a final conclusion in this regard, so future studies might pick up these possibilities and specifically focus on the relation between the different processing stages of online advertisements, particularly from attentional effects of demographically targeted ads to ad evaluation and its subsequent transfer to brand evaluation, as well as potential generalization effects to the whole website at the end. The present study provides a first explorative basis for future research in this direction.

In the present study, demographic targeting did not affect the evaluation of the website. We may speculate that internet users usually consider online advertisements as rather independent entities which are rarely perceived as an inherent part of the original website. This assumption is supported by the findings of Rieger et al. [45], who even found that the congruency between ad content and the editorial content of webpages did not have an effect on the users’ attitude towards the overall website. But, our assumption is at odds with results of some previous studies showing transfer effects from website elements to website evaluation [36]. We hence conclude that current knowledge is too sparse to comprehensively understand the mechanism of such transfer phenomena, while the present study indicates that attention for personally relevant advertisements, although being a necessary condition for subsequent judgment formation according to the model of human information processing [10], is not sufficient to elicit positive effects on the level of subjective judgments.

### Internal and external validity of the present study

The design of the present study strictly followed the design used by Köster et al. [19] to permit a comparison of the effect sizes found in the two studies that mainly differed with respect to the task the participants performed during webpage observation. That is, demographic targeting was based on the same demographic variables, leading to a comparable sample of participants, and the spatial configuration of the ads were identical. The present study was a true experiment with randomization of subjects to experimental groups (demographically targeted versus non-targeted ads). Edgington [62] highlighted that randomization "will not permit statistical inferences about persons not used in the experiment but it will permit statistical inferences about treatment effects for the experimental subjects" (p.485). Correspondingly, the present effect of demographic targeting on visual attention is based on a study with high internal validity, as randomization makes groups comparable according both known and unknown factors that may act as confounding variables (e.g., differences in participants’ interest to explore webpages spatially extensively [16]). Moreover, we also minimized potential biases between demographically targeted versus non-targeted ads on the level of image statistics by means of two saliency computation models [52, 53] that were used to iteratively create two versions of each ad which clearly differ regarding the degree of demographic targeting while image statistics were matched. Thus, the differences in attention allocation found between demographically targeted and non-targeted ads was solely driven by the ads’ semantic content (reflecting the degree of personal relevance) but not by differences on the level of basal image statistics and not by systematic differences between the two groups of participants.

However, as emphasized by Lang [63], “the results of experiments cannot be statistically generalized to larger populations” (p. 427), limiting the external validity of the present study. For example, we only included German female students in our sample and hence cannot make conclusions about the effect sizes expected in men or in other cultures. Indeed, a focus on cross-cultural differences appears to be a fruitful avenue for future research. Cross-cultural differences in the perception of facial emotions [64], geometric properties [65], complex scenes [66], and even webpages [67] have already been reported. Moreover, external validity may be also limited to the specific webpages and ads used in the present study. The type of ad configuration was specifically selected in order to allow a direct comparability with the ad type used by Köster et al. [19], representing the standard format of the website of Germany’s most sold newspaper (Bild Zeitung). However, Owens et al. [68] found that banner blindness was more pronounced when the ad, in accordance with the skyscraper banner used in the present study, was placed on the right side of the webpages. It is conceivable that demographically targeted ads are even more effective when placed at other locations. Also, animated banner ads, compared to the static ads used here, usually attract more attention [7]. With respect to future studies, we must admit that it is hardly possible to examine all potential design configurations of ads in one single study. This circumstance significantly reduces the external validity of every study. Hence, the main focus should be directed to internally valid study designs enabling the assessment of the effectiveness of individual design factors, whereas the major tool employed for generalization purposes “is replication–specifically, the replication of experimental results with many different specific subjects in many different specific locations” ([63], p. 429).

### Implications for future studies

Besides the need for replication studies with different sample characteristics and demographic properties, the adequate control for differences in image statistics between experimental conditions, as well as different visual designs of webpages and ads, we want to point out three further implications for future research derived from the present study:

First, studies that focus on the effect of personalized or customized ads on visual attention have to deal with the problem of negative user reactions. Negative effects in the observer such as irritation, reactance, or avoidance behavior are particularly likely when personalization is realized by means of the incorporation of the individual’s personal data into the ad. In this case, privacy concerns increase [22, 23] and may lead, inter alia, to reduced purchase intentions [21]. In contrast, demographic targeting may trigger privacy concerns rather rarely. On the negative side, this methodological account may lead to a reduced fit between the advertised brands and the actual needs of the individual internet user. In the present study, we therefore used and averaged across a relatively heterogeneous sample of brands and associated product classes in order to counterbalance potential inter-individual differences in participants’ pre-study product involvement. This procedure is in line with the common procedure used in vision research (cf. [69]), where participants observe rather large sets of visual stimuli to counterbalance both inter-individual differences in the perceived interestingness of the stimuli which usually affect viewing behavior in a top-ton manner [16] as well as the variance of visual properties which vary across images [70]. Hence, we recommend not to limit the stimulus material to only one or few stimuli when examining online advertising.

Second, and related to this: When using real brands or product classes, as in the present study, controlling for brand/product familiarity and prior attitudes towards the brand/product is a challenging task. Some authors therefore use fictitious brands and products, but these usually suffer from ecological validity and may produce artificial results as well. We did not assess brand familiarity and prior attitude in the main study due to two reasons: First, prior presentation of target elements, such as brand logos and images of products, would have primed participants’ visual attention and hence would have substantially biased the primary effect we were interested in. Second, an assessment of these two variables after stimulus presentation in the eye-tracking session would have biased this follow-up measurement. At least, we can conclude that participants’ overall attitude towards the selected brands was moderate (no ceiling effects). Future studies may focus on this aspect in more details. Nonetheless, the methodological challenge is tough as a measurement of brand attitude and familiarity prior to the eye-tracking part distorts ad effects on overt attention when being temporally too close (priming effects) or the validity of such a measurement is threatened when it is temporally too far away (e.g., one week or month before) as participants could make further product experiences in the meantime. In so far, a profound methodological solution for this problem would be desirable for many studies in the present research field.

Finally, eye-tracking techniques capture automatic consumer information when interacting with websites, thus becoming a new and more interesting tool (than previous self-reports) to evaluate consumers’ performance. The results of the current study could be corroborated by using tools that capture distinct and moment-by-moment consumer reactions in online environments, as fMRI [71] or noninvasive sensors measuring heart rate and skin conductance [72]. More broadly, several methodologies exist to overcome limitations of traditional self-reports, which is an important step towards a profound analysis of all the complete processing stream including attention, memory, attitudes, judgments, and behavioral responses to online ads.

## Supporting information

S1 Data set The SPSS statistics data document (.sav) with eye tracking data, brand evaluation data, and website evaluation data. (SAV)
